# Construct Clarity in Physician-Hospital Alignment: The Need for Precision in Definition, Measurement, and Management: Comment on "Alignment in the Hospital-Physician Relationship: A Qualitative Multiple Case Study of Medical Specialist Enterprises in the Netherlands"

**DOI:** 10.34172/ijhpm.8644

**Published:** 2024-10-02

**Authors:** Chad T. Brinsfield

**Affiliations:** Management Department, Opus College of Business, University of St. Thomas, Saint Paul, MN, USA.

**Keywords:** Hospital-Physician Relationships, Physician-Hospital Alignment, Construct Clarity, The Netherlands

## Abstract

The study by Ubels and van Raaij highlights the importance of alignment in hospital-physician relationships and the challenges in understanding, measuring, and managing it. Despite extensive research on alignment, drawing precise conclusions about its nature, drivers, and outcomes is difficult due to construct clarity and construct validity issues. This commentary focuses on clarifying these issues and the problems they create for hospitals attempting to manage alignment, as well as for scientific inquiry in this area. These issues involve the need to specify more clearly the essential nature of alignment and how it is distinct from other constructs such as engagement. It also involves demarcating alignment from the structures, arrangements, or processes intended to foster it, as well as from its outcomes. Improved precision in these areas will enable the development of more reliable and valid measures, thereby supporting hypothesis testing, theory building, and the identification of best practices.

## Introduction

 The recent article by Ubels and van Raaij^1^ provides an in-depth examination of alignment within hospital-physician relationships through five case studies in the Netherlands. Their research takes place in the wake of healthcare reforms enacted by the Dutch government in 2015 that were intended to increase alignment between hospitals and physicians. These reforms had the unintended effect of prompting physicians to form medical specialist enterprises (MSEs), uniting physicians within a hospital into a single representative unit. By forming MSEs, physicians sought to protect their interests and ensure a high level of patient care.

 Ubels and van Raaij^1^ used a multi-method approach to assess alignment between the hospital and the MSE, which entailed contract analysis, and interviews with board members of both entities. They focused on three components of alignment: (1) Strategic alignment, which involves sharing a common mission, vision and strategy and working together to accomplish the mission; (2) Financial alignment, which refers to the extent to which business models, costs, risks, and financial incentives are aligned and shared; and (3) Alignment among physicians, which assesses how well the interests of all doctors are represented by the MSE.

 Ubels and van Raaij^1^ examined governance style as a determinant of alignment, distinguishing between contractual and relational types. Contractual governance relies on formal, legally binding agreements detailing duties, obligations, and penalties. In contrast, relational governance is based on informal, trust-based relationships that emphasize mutual interests and collaboration. They found that hospitals and MSEs relying more on relational governance reported higher levels of alignment compared to those using contractual governance.

## Issues of Construct Clarity and Validity

 The study by Ubels and van Raaij^1^ underscores the importance of alignment in hospital-physician relationships and also highlights the complexity involved in studying and understanding it. The concept of employee-organization alignment has been of interest to management scholars for over 100 years. In the past 30 years alignment between physicians and hospitals has become a topic of interest in health care management due to increasing integration of physicians into hospital operations and finances. Alignment is important because people are inherently self-interested and alignment leverages this in accordance with the interests of the organization. This is a central premise of agency theory,^2^ which Ubels and van Raaij^1^ draw on as a primary theoretical framework in their study. Agency theory explains the relationship between principals (eg, hospitals) and agents (eg, physicians), focusing on how the principal can incentivize the agent to act in the principal’s interest, while managing the risk of opportunistic behavior. Although agency theory has primarily emphasized economic self-interest, Ubels and van Raaij^1^ note that non-economic psychosocial considerations also play a role in shaping perceptions of alignment and behavior. They draw on the lesser-known social theory of agency^3^ to help explain these aspects of alignment.

 Practitioners and management theorists are concerned with alignment because it serves as a key mediating mechanism in achieving desired outcomes. Although in the Ubels and van Raaij^1^ study alignment is the dependent variable with the inference that alignment leads to ultimate outcomes of interest such as improved patient care and better financial performance. Understanding mediating factors is crucial because they help clarify the underlying causal mechanisms, essentially explaining how and why certain management practices lead to specific results. For instance, understanding that alignment bridges structural arrangements (like governance style or employment), or human resource practices (like pay-for-performance), with outcomes such as patient care and financial performance, enables managers to implement more targeted approaches to achieve desired results.

 Although there is a substantial body of research on alignment,^4^ drawing precise or generalizable conclusions about its nature, drivers, and associated outcomes is challenging. Furthermore, while most hospital administrators acknowledge the importance of alignment, few actually measure it. Among those who attempt to measure it, it is often unclear whether they are truly measuring alignment or something else. This same issue often applies to research in this area. These challenges stem from a lack of clarity and consistency in defining the construct, leading to issues with construct validity.

 When alignment is clearly defined and understood, organizations can accurately measure and track the effects interventions designed to improve alignment actually have on alignment. Similarly, they can track the effects alignment has on outcomes such as financial results, patient outcomes, and physician well-being. This more precise understanding of the organization’s dynamics enables more targeted and effective corrective measures, rather than generalized solutions that may not address the root causes of problems. Conversely, without clear and consistent definitions and valid metrics, it is difficult to accurately assess where and how misalignments occur. This ambiguity complicates the accurate identification of problems because issues related to culture, leadership, strategy, operational inefficiencies, perceptions of fairness, trust, or engagement, while potentially related to alignment, are distinct and likely require different approaches to address their unique causes and consequences.

 Ubels and van Raaij^1^ adopt the definition of alignment as “the degree to which physicians and organized delivery systems share the same mission and vision, goals and objectives, and strategies, and work toward their accomplishment” (as cited in Shortell et al,^5^ p. I-2; Ubels and van Raaij,^1^ p. 3). In this definition, the essential nature of alignment is encapsulated in the phrase ‘share the same.’ The principals in alignment are physicians and organized delivery systems. The factors being aligned include mission, vision, goals, objectives, and strategies. The inclusion of the phrase “work toward their accomplishment” suggests that alignment is not merely a structural arrangement or a psychological state, but also requires active collaboration and effort. However, this phrase complicates the operationalization of alignment, as it introduces a dynamic element that is influenced by factors beyond alignment itself, potentially blurring the distinction from other constructs. Hence ‘work toward their accomplishment’ is better viewed as an outcome of alignment rather than a defining characteristic.^6^

 Ubels and van Raaij’s^1^ method for evaluating alignment shares elements with this definition’s components, but their assessment extends well beyond the definition (See Ubels and van Raaij’s^1^ Supplementary files 1 and 2). Their expansive and multi-method assessment considers factors such as quality policies, participation, opportunism, types of incentives, conflict resolution, and more. This approach highlights the intricate nature of managing alignment and the numerous organizational factors influencing it or resulting from it. However, the complexity of managing alignment, including its interrelationships with a wide array of organizational factors, should not be mistaken for the complexity of the phenomenon itself. When a management concept becomes overly broad, discriminant validity is compromised, making it challenging to identify the precise nature of related problems, leading to overgeneralized solutions and misallocation of resources.

 Further complicating the advancement of scientific inquiry, the definition used by Ubels and van Raaij^1^ is just one of several found in the literature, which vary in terms of the essential nature of alignment, the factors being aligned, and the basis for operationalization (See Brinsfield et al^6^; Burns et al^4^). A review of these definitions raises important questions: Is “sharing the same” the core essence of alignment? Are mission, vision, goals, objectives, and strategies the requisite alignment factors? Are these factors clearly defined, comprehensive, and parsimonious? Can alignment be objectively measured, or should it be based on perception? Can alignment be accurately assessed using a Likert-type scale, or is it too complex or context-dependent for such standardized measurement? Should alignment be considered a standalone construct, or is it better understood as being indicated by the presence of other structures, processes, established constructs, or outcomes?

## Pathways to Improved Construct Clarity

 These questions each need not have a singular answer, especially as scholars map out the conceptual terrain and try to understand the complexity and perspectives that alignment may encompass. However, this variability should be acknowledged and systematically managed if scientific inquiry in this domain is to proceed efficiently. At a minimum, some fundamentals of construct clarity are essential.

 For instance, more consistent use of terminology is needed. Due to the multidisciplinary nature of alignment (eg, strategy, psychology, healthcare management, and organizational behavior) people study distinctly different phenomena but call it the same thing, or study practically equivalent phenomena but talk past each other because they are using different terminology. Terms like integration, employment, fit, congruence, relationships, line of sight, have all been used to describe alignment. Effective communication is facilitated when researchers, practitioners, and policy-makers share a common understanding of key terms and concepts.

 Both objective alignment and perception of alignment may be useful, but it’s important to understand the rationale and trade-offs of each approach. In the Ubels and van Raaij^1^ study, they analyzed contracts, which could represent objective alignment, though these factors can be perceived differently. They also interviewed hospital administrators and physician board members of the MSEs. By combining contractual analyses with interviews, they formed an aggregate measure of alignment. This approach highlights the complexity of measuring alignment, as in this example, three different sources are combined to form the aggregate measure, but each of these sources is distinct and has distinct causes and consequences.

 Furthermore, efficiently implementing this approach while ensuring reliability and scalability presents significant challenges for hospitals and researchers. Methods such as interviews and contract reviews, while rich in detail, are resource-intensive and typically more subjective compared to validated Likert-type scales. Reflecting this limitation, Ubels and van Raaij^1^ conducted only two interviews per hospital, potentially missing a fuller spectrum of perspectives within each organization. In contrast, Likert-type measures allow for more frequent and scalable assessments, which could help hospitals detect misalignments sooner and capture a broader range of perspectives, enabling more timely and effective interventions. These measures also would provide more reliable data for hypothesis testing and support theoretical development. This would facilitate cross-study comparisons, and the establishment of benchmarks and best practices.

 Creating reliable and valid measures necessitates clearly articulating the essential nature of alignment, identifying the principals and factors being aligned, and explicating its basis for operationalization. It also requires distinguishing alignment from other constructs that may be antecedents, correlates, or outcomes of alignment, but are not alignment itself, such as pay-for-performance, risk sharing, employment, commitment, trust, engagement, motivation, or performance. In an effort to advance construct clarity, Brinsfield et al^6^ defined physician-hospital alignment as “a physician’s perception that their financial incentives, goals, and values, and those of their hospital, are mutually supporting and reinforcing, rather than in conflict with one another.” This definition specifies the essential nature of alignment, clearly identifies the principals and factors being aligned, and explicates the basis for operationalization as a physician’s perception. This definition sets the stage for the development of valid and reliable measurement.

 Alignment is likely more complex than any one definition or perspective can fully capture. For instance, alignment is important at varying levels of analysis, such as multi-level (ie, between a hospital and a physician), or individual level (ie, between a physician’s own financial incentives and their values or goals), or intra-organizational level (ie, between a hospital’s own financial incentives and its values or goals). Thus, alignment can take many different forms and be examined through various trajectories and relationships. Whatever form alignment takes, it should be clearly defined and distinctly demarcated from other constructs.

 Advancement in management science is an iterative process, evolving as new insights and methodologies emerge. While perfection is unrealistic, continuous improvement in key areas is essential. Achieving construct clarity is fundamental, requiring the creation of precise definitions that distinctly differentiate concepts like alignment from related constructs such as engagement, as well as from its antecedents and outcomes (such as governance and performance). Such clarity is essential not only for the development of reliable and valid measures which are critical for hypothesis testing and theory building, but also for facilitating effective communication and collaboration among researchers, practitioners, and policy-makers. By focusing on these priorities, we can advance scientific inquiry, improve the practical application of research, and enhance organizational performance and patient care outcomes in healthcare.

## Ethical issues

 Not applicable.

## Conflicts of interest

 Author declares that he has no conflicts of interest.
